# Sustainable PLA Composites Filled with Poaceae Fibers: Thermal, Structural, and Mechanical Properties

**DOI:** 10.3390/ma18173952

**Published:** 2025-08-23

**Authors:** Natalia Kubiak, Bogna Sztorch, Magdalena Kustosz, Miłosz Frydrych, Daria Pakuła, Marek Jałbrzykowski, Tobias Hartmann, Camilo Zopp, Lothar Kroll, Robert E. Przekop

**Affiliations:** 1 Faculty of Chemistry, Adam Mickiewicz University in Poznań, Uniwersytetu Poznańskiego 8, 61-614 Poznań, Poland; natkub8@amu.edu.pl (N.K.); magdalena.kustosz@amu.edu.pl (M.K.); 2 Centre for Advanced Technologies, Adam Mickiewicz University in Poznań, 10 Uniwersytetu Poznańskiego, 61-614 Poznań, Poland; frydrych@amu.edu.pl (M.F.); darpak@amu.edu.pl (D.P.); 3 Faculty of Mechanical Engineering, Bialystok University of Technology, Wiejska 45C, 15-351 Bialystok, Poland; m.jalbrzykowski@pb.edu.pl; 4 Cluster of Excellence MERGE, Chemnitz University of Technology, 09107 Chemnitz, Germany; tobias.hartmann@mb.tu-chemnitz.de (T.H.); camilo.zopp@mb.tu-chemnitz.de (C.Z.); lothar.kroll@mb.tu-chemnitz.de (L.K.)

**Keywords:** PLA, natural fibers, injection molding, Poaceae fibers, biocomposites

## Abstract

The present study investigates the manufacturing and characterization of poly(lactic acid) (PLA)-based composites with raw and treated Poaceae, with loadings of 5, 10, and 20% wt. Before composite fabrication, the lignocellulosic fillers were analyzed using Fourier transform infrared spectroscopy (FTIR), thermogravimetric analysis (TGA), and microscopy to assess their chemical composition, thermal stability, and morphological features. Composites were prepared by melting PLA in a molten state with fillers, followed by injection molding. Comprehensive characterization of the obtained composites included microscopic analysis, melt flow index (MFI) testing, and differential scanning calorimetry (DSC), as well as mechanical tests (tensile and bending tests, impact test). The addition of Poaceae fibers to the PLA matrix significantly affected the mechanical and rheological properties of the composites. Incorporating 5% of cooked or alkalized fibers increased the flexural strength by 57% and 54%, respectively, compared to neat PLA. The modulus of elasticity for the composite with 20% alkalized fibers increased by as much as 35%. The fibers acted as nucleating agents, reducing the cold crystallization temperature (T_cc_) by up to 15.6 °C, while alkaline residues contributed to an increased melt flow index (MFI). The conducted research provides a valuable basis and insights into the design of sustainable bio-based composites.

## 1. Introduction

The emergence of synthetic polymers marked the beginning of a profound technological revolution, fundamentally reshaping numerous aspects of contemporary life. Due to their exceptional properties, plastics rapidly became highly desirable materials, finding widespread application across virtually every sector of human activity [1]. In response to this growing demand, large-scale industrial production was initiated. However, conventional plastics are predominantly derived from petroleum-based resources, which are inherently finite and are being depleted at an accelerating pace. In addition to concerns over resource scarcity, the accumulation and disposal of plastic waste at the end of product life cycles present a major environmental challenge [2]. As a result, plastics have become a pressing global issue that must be addressed, particularly in the context of the escalating climate crisis. Recently, biopolymers derived from renewable resources have emerged as a promising alternative to traditional petrochemical-based plastics [3]. A key advantage of biopolymers lies in their ability to exhibit properties comparable to those of conventional plastics while also being fully biodegradable under composting conditions without releasing toxic byproducts. Polylactic acid or poly(lactic acid) (PLA) has garnered the most significant attention in academic research and industrial applications due to its favorable material properties, processability, and commercial viability [4,5]. PLA is a thermoplastic biopolymer belonging to the family of aliphatic polyesters. It is synthesized from lactic acid, which exists in two stereoisomeric forms—*L*- and *D*-lactic acid. PLA is fully biodegradable; however, its degradation occurs only under industrial composting conditions, which require controlled temperature, humidity, and pH to facilitate the breakdown process [6,7]. PLA offers several advantages, including mechanical properties comparable to conventional petrochemical-based polymers, ease of processing using standard manufacturing techniques, dimensional stability, and high optical clarity. These characteristics make PLA a suitable candidate for the production of items with performance profiles similar to those made from non-renewable sources. Among the limitations that currently restrict the broader use of PLA are its relatively low impact resistance and limited elongation at break, both of which require further material optimization [8]. To overcome these limitations, researchers have explored a variety of modification strategies. These include copolymerization with other monomers during synthesis [9], the formulation of polymer blends [10], the incorporation of nucleating agents to enhance crystallization [11], and the addition of plasticizers such as citrates, poly(ethylene glycol) (PEG), and poly(propylene glycol) (PPG) [12,13]. Mechanical properties can also be modified through the incorporation of additives and fillers, which not only enhance the mechanical and thermal performance of the resulting composites but also enable control over their biodegradation behavior. The use of natural fillers—especially those derived from industrial waste streams—offers an additional advantage by significantly reducing production costs and repurposing process residues, thereby granting them new functionality. This approach can substantially extend the life cycle of the materials and effectively reduce the volume of waste generated. The current environmental crises have stimulated research into biodegradable composites as a sustainable alternative to synthetic materials. However, the production of biopolymer composites from agricultural waste poses technological challenges that require innovative solutions. According to the latest reports, the biggest problems encountered in this field include the need to use specialized methods for fiber extraction and processing [14,15]. Another challenge is ensuring strong adhesion between the biopolymer matrix and the reinforcements derived from agricultural waste, as differences in the surface chemistry and morphology of these materials can hinder effective bonding. Another important limitation is the low maximum processing temperature of natural reinforcements, as the cellulose molecules contained in plant fibers usually degrade at temperatures between 180 °Cand 200 °C. Despite a large number of studies on surface modification techniques and improved properties of biocomposites, further research and new innovations are needed to implement more cost-effective and environmentally friendly methods [16,17].

However, the incorporation of fillers into polymer matrices presents a key challenge: the limited interfacial adhesion (i.e., physical interactions) between the filler surface and the polymer matrix [18]. The incorporation of natural fillers not only affects its mechanical and structural properties but also significantly influences rheological behavior. One of the main key parameters of processability is MFI. While a higher filler content typically increases system viscosity, some studies report reduced viscosity due to shear-thinning effects [19,20]. The surface characteristics and chemical composition of the fibers play an important role in this context. For instance, alkaline treatment may smooth the fiber surface and remove amorphous components, which can promote fiber alignment during flow and reduce internal friction. These effects, including the so-called “ball-bearing” phenomenon [21] or wall slip at low shear stresses [22,23], can lead to increased melt flow rates. Understanding how fiber modification alters the flow behavior of PLA-based composites is crucial for tailoring their processing characteristics and end-use performance.

The mechanical performance of PLA composites reinforced with natural fibers is highly dependent on the filler type, content, and surface treatment. Previous studies have shown that while the addition of such fibers often leads to a reduction in tensile strength at higher concentrations, low filler loadings—especially after alkaline treatment—can improve mechanical performance due to better stress transfer and enhanced fiber dispersion [24,25]. However, excessive filler content promotes agglomeration, which may act as structural defects, reducing composite integrity. Other critical factors include fiber morphology, diameter, and surface composition (e.g., lignin, waxes), which strongly affect interfacial adhesion with PLA [26]. Furthermore, the presence of nucleating agents within treated fibers may locally increase crystallinity and material brittleness [27]. These factors collectively underscore the need for careful optimization of filler characteristics and processing conditions to achieve a balanced enhancement in composite properties.

Besides tensile properties, flexural strength and modulus are also critically influenced by fiber content and treatment. Natural fibers generally increase the stiffness of composites, leading to improved resistance to bending loads, although excessive filler content can deteriorate flexural strength due to matrix discontinuity and fiber clustering [28,29].

The use of natural fillers may open new horizons for sustainable and eco-friendly industries, particularly when their mechanical properties are comparable to those of conventional materials, thereby enabling their substitution [30].

The most widely used include sunflower husk [31,32,33,34,35,36], hemp fibers [37,38,39,40,41,42], reed [43,44,45,46], jute [47,48], coconut fibers [49,50,51,52,53], and wood fibers [54,55,56]. Poaceae has also emerged as a promising, albeit currently rarely used, filler material. It is an inexpensive and widely available resource found on all continents except Antarctica. Botanically, Poaceae belongs to the group of angiosperms and is classified within the Poaceae family. Poaceae fibers are primarily composed of cellulose, hemicellulose, and lignin. This study presents the effect of Poaceae fiber addition and processing methods on the physicochemical properties of PLA-based composites.

The research aimed to evaluate the potential of natural plant fibers as environmentally friendly reinforcements for polymeric materials. A comprehensive set of analyses were conducted, including thermal analysis (DSC, TGA), spectroscopic analysis (FT–IR), microscopic observations, and extensive mechanical testing, such as tensile, flexural, and impact strength measurements. Additionally, surface property assessments and melt flow index (MFI) evaluations were performed to determine the processability of the composites. The obtained results enabled the assessment of the influence of Poaceae fiber type and treatment on the structure, mechanical performance, and processing behavior of PLA composites, highlighting directions for further research into using renewable raw materials in materials engineering.

## 2. Materials and Methods

### 2.1. Materials

Polylactide (PLA) type Ingeo 2003D was purchased from NatureWorks LLC (Minneapolis, MN, USA). The Poaceae (grass) in pellets was purchased from the ARDEX Wytwórnia Pasz (Sochaczew, Poland). The Poaceae family, also known as grasses, is one of the most important plant families in Poland and worldwide, comprising over 150 naturally occurring species, as well as numerous cultivated and naturalized ones. These species form the main component of grassland, meadow, and pasture vegetation and include key cultivated plants such as cereals.

### 2.2. Fiber Processing

The raw material in the form of pellets was subjected to grinding with a SHINI SG-1417–CE (ShiniEurope, Częstochowa, Poland) slow-speed knife mill, followed by fractionation of the material with a vibrating screen. After the process, a material fraction of 250–1000 μm was obtained, which was used to prepare composites.

#### 2.2.1. Alkaline Maceration with Potassium Hydroxide

The procedure involved adding the raw plant material and potassium hydroxide in a weight ratio of 1:5 (calculated on a dry weight basis) into a container. Demineralized water was then added to thoroughly moisten the mixture. The entire content was stirred until a homogeneous mass was obtained. To ensure complete hydrolysis, the mixture was left to incubate for 10–15 days. After incubation, the mixture was transferred onto a sieve lined with a cotton filter and rinsed thoroughly with demineralized water until a neutral filtrate was achieved. The resulting material was then dried at 55 °C.

#### 2.2.2. Boiling

In this procedure, the raw material was combined with a 12.5-fold excess of demineralized water in a container and heated to the boiling point for 2 h. The mixture was then filtered through a sieve lined with a cotton filter. The filtrate was discarded, and the solid material was returned to the container for further boiling for another 2 h. This process was repeated until a clear filtrate was obtained (typically 5 to 10 repetitions). The final residue was dried at 55 °C.

### 2.3. Preparation of Granulate

PLA 2003D and Poaceae were homogenized using a laboratory two-roll mill ZAMAK MERCATOR WG 150/280 (Zamak, Skawina, Poland). A portion of 800 g PLA and 200 g of Poaceae (raw, cooked, and alkalized) was mixed until the final concentration of the Poaceae was 20%. The mixing was performed at a roll temperature of 210 °C for 10 min to achieve full homogeneity. The masterbatch was granulated with a SHINI SG-1417-CE grinding mill and then dried for 24 h at 60 °C.

### 2.4. Injection Moulding Process

**Figure 1:**
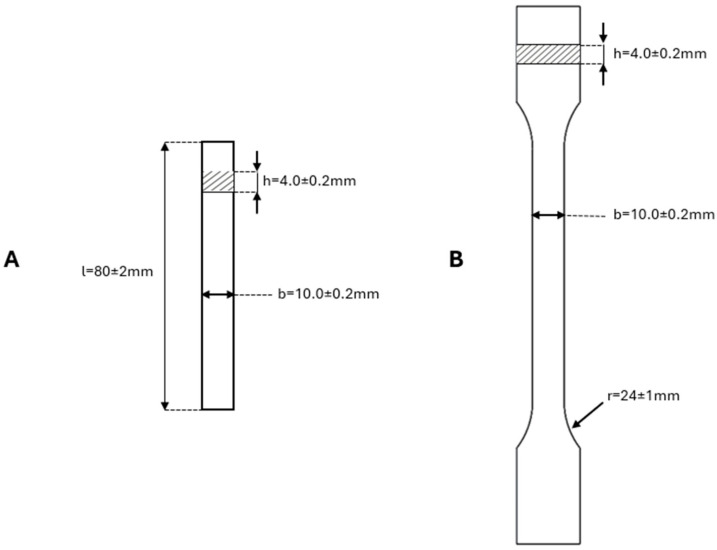
Dimensions of samples used for the mechanical tests: (**A**) flexural and (**B**) tensile tests.

The prepared masterbatches were diluted to obtain final Poaceae concentrations of 5, 10, and 20% wt. with PLA directly in an Engel E-victory 170/80 injection molding machine (ENGEL, Schwertberg, Austria)with the following zone temperatures: nozzle 180 °C, Zone 1 170 °C, Zone 2 175 °C, Zone 3 175 °C, and feeder 40 °C. A holding pressure with a linear increment over time was applied. The mold temperature was maintained at 20 °C. Standardized specimens for mechanical tests were obtained according to PN EN ISO 20753:2019-01 (Figure 1) [57]. Final systems concentrations were 20, 10, and 5% wt. for raw, cooked, and alkalized Poaceae. The injection molding parameters were as follows: clamping force 800 kN, holding pressure profile 1000 bar, holding time 10 s, cooling time 40 s, and screw diameter 25 mm.

### 2.5. Analysis

Fourier transform infrared (FTIR) spectra were recorded on a Nicolet iS50 Fourier transform spectrophotometer (Thermo Fisher Scientific, Waltham, MA, USA) equipped with a diamond ATR unit with a resolution of 0.09 cm^−1^.

Thermogravimetry (TGA) was performed using a NETZSCH 209 F1 Libra gravimetric analyzer (Netzsch, Selb, Germany). Samples of 8 mg ± 0.5 mg were placed in Al_2_O_3_ crucibles. Measurements were conducted under nitrogen and air (flow of 20 mL/min) in temperature ranges from 25 °C to 1000 °C and at a 10 °C/min heating rate.

Differential scanning calorimetry (DSC) was performed using a NETZSCH 204 F1 Phoenix calorimeter (Netzsch, Selb, Germany). Samples of 6 ± 0.2 mg were placed in an aluminum crucible with a punctured lid. The measurements were performed under nitrogen at temperatures from 20 to 220 °C and at a 10 °C/min heating rate.

The Poaceae and surface structure were analyzed under a Keyence VHX 7000 digital light microscope with a 100× to 1000× VH–Z100T lens (Keyence, Osaka, Japan). All of the pictures were recorded with a VHX 7020 camera.

For impact strength tests, the obtained materials were injection-molded into dumbbell specimens of type 1B under PN-EN ISO 527-1:2020-01 [58] and PN-EN ISO 178:2019-06 [59]. A Charpy impact test (with no notch) was performed on an Instron Ceast 9050 impact machine using rectangular specimens (80 mm × 10 mm × 4 mm).

Tensile and flexural tests of the obtained specimens were performed on a universal testing machine, INSTRON 5969 (Instron, Norwood, MA, USA), with a maximum load force of 50 kN. The traverse speed for the tensile strength measurements was set at 2 mm/min, and for the flexural strength, it was also at 2 mm/min. The flexural tests were carried out following PN–EN ISO 178 [59], while the tensile tests adhered to PN–EN ISO 527 [58].

The effect of the fiber addition on the mass flow rate (MFI) was also determined. The measurements were made using an Instron plastometer, model Ceast MF20 (Instron, Norwood, MA, USA), according to the applicable standard PN-EN ISO 1133:2006 [60]. The measurement temperature was 210 ± 0.5 °C, while the piston loading was 2.16 kg.

The molecular weight distribution of the samples was analyzed using gel permeation chromatography (GPC) on an Agilent 1260 Infinity system equipped with a Phenogel 10 µm Linear (2), 300 × 7.8 mm column. The samples were dissolved in tetrahydrofuran (THF), which served as the eluent. The measurement conditions were as follows: analysis time 15 min, eluent flow rate 1.0 mL/min, column temperature 35 °C, and refractive index (RI) detector temperature 35 °C. Molecular weights and polydispersity indices (PD) were determined relative to a calibration curve obtained using linear polystyrene standards with molecular weights ranging from 1000 to 3,500,000 g/mol.

## 3. Results

### 3.1. Filler Analysis

#### 3.1.1. FTIR Analysis

**Figure 2:**
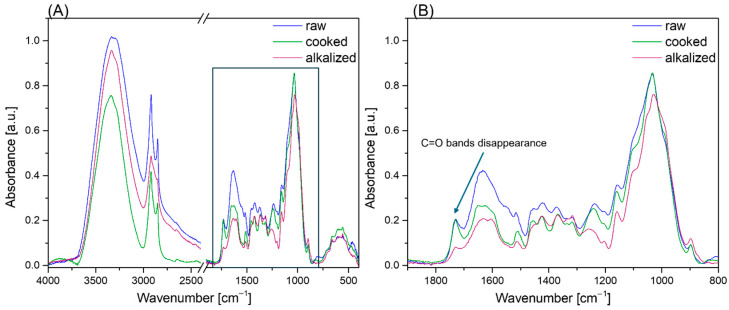
FTIR spectra: (**A**) full range, (**B**) detailed changes.

Characteristic absorption bands of the individual components of the fibers were determined using FTIR in attenuated total reflectance mode (Figure 2). In the spectra of the unmodified compounds, an intense band at 3334 cm^−1^ was observed, corresponding to the stretching vibrations of –OH groups present in cellulose I or α–cellulose [61]. The bands at 2918 cm^−1^ and 2850 cm^−1^ were assigned to C–H stretching and CH_2_ bending vibrations, respectively [62]. The stretching and bending vibrations of C = O groups, present in lignin and hemicellulose, were identified at 1729 cm^−1^. The bands at 1456 cm^−1^, 1364 cm^−1^, 1317 cm^−1^, and 1222 cm^−1^ were attributed to the bending vibrations of C-H, O-H, or CH_2_ groups. Absorption at 1030 cm^−1^ was assigned to the stretching vibrations of C–O bonds in cellulose and hemicellulose. For the samples subjected to alkali maceration, the band at 1729 cm^−1^, attributed to the C = O stretching vibrations of carboxylic acid or ester groups, disappeared as a result of hemicellulose and lignin partial removal [63]. Furthermore, the intensity ratio of the absorption bands at 1723 cm^−1^ to 1030 cm^−1^ was calculated, yielding values of 0.043 for the untreated samples, 0.035 for the boiled samples, and 0.0057 for the alkali-treated samples.

Additionally, the band corresponding to the C = O stretching vibrations of the acetyl group in lignin, located at 1243 cm^−1^, showed lower intensity compared to the unmodified and boiled samples. The conducted analyses indicate that alkali treatment leads to the partial removal of lignin and hemicellulose, which enables better wetting of the fibers by the polymer matrix, which directly affects the quality of the bond between the fiber and the composite matrix.

#### 3.1.2. TGA Analysis

**Figure 3:**
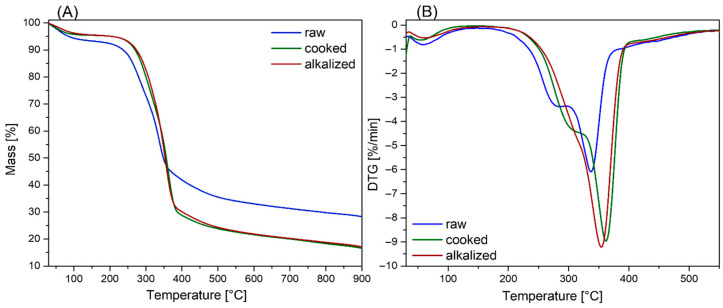
TGA analysis in nitrogen: (**A**) mass change, (**B**) DTG.

The results of thermogravimetric analysis conducted on the investigated samples revealed the presence of three main stages of thermal degradation, as shown in Figure 3 and Table 1. The first stage, occurring in the temperature range of 55–60 °C, is characterized by a minor mass loss of approximately 3–8%, which is primarily attributed to the removal of physically adsorbed moisture and low-molecular-weight volatiles naturally present within the material’s structure. The second principal stage of thermal decomposition is observed within the temperature range of 180–420 °C and is associated with the degradation of carbohydrate components, primarily hemicellulose and cellulose. This stage exhibits the greatest total mass loss, ranging from 60% to 75%, and results in the emission of low-molecular-weight pyrolysis products, including carbon oxides, water vapor, and volatile organic compounds [64]. The third degradation stage, occurring above 420 °C, corresponds to the slow and incomplete breakdown of lignin, an amorphous, aromatic biopolymer with a broad range of thermal stability. Furthermore, samples subjected to chemical modification displayed a shift in the temperatures corresponding to the maximum degradation rates toward higher values. This phenomenon is interpreted as a result of the reduced relative content of hemicellulose and lignin in the fiber structure, induced by the applied treatment process. These findings are consistent with the FTIR analysis, which showed a decrease in the intensity of characteristic absorption bands corresponding to lignin and hemicellulose (Figure 2). Additionally, in the case of samples subjected to alkalization, the absence of two distinct peaks in the 200–400 °C range suggests that KOH treatment led to a more efficient removal of amorphous components compared to cooked samples. This may indicate a more thorough extraction of alkali-soluble substances, such as hemicelluloses and lignin fragments. Moreover, the highest residual mass was observed for the raw Poaceae sample, indicating a higher retention of thermally recalcitrant substances that were not extracted during processing, in comparison to the treated samples.

#### 3.1.3. Microscopy

**Figure 4:**
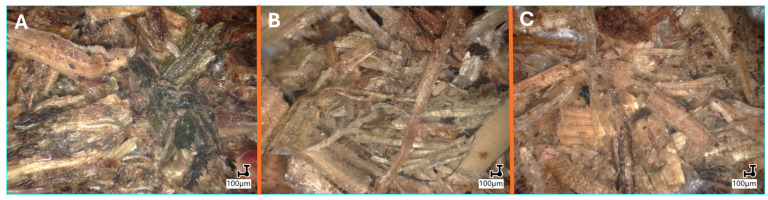
Microscopic images illustrating the fibers used: (**A**)—untreated (raw), (**B**)—thermally treated (cooked), (**C**)—chemically treated (alkalized).

Figure 4 shows microscopic images of Poaceae samples before and after thermal (cooking) and chemical (alkalinization) treatment. Both modification methods affect the structure of the fibers in a different way, which is reflected in their physicochemical properties. Alkalinization leads to the selective removal of specific components, such as hemicelluloses, lignin, or oily substances, while cooking mainly causes the disintegration of cell structures and the washing out of soluble components.

The treatment also induces noticeable changes in the color of the fibers (Figure 4B,C) when compared to the untreated reference sample (Figure 4A). In particular, the alkaline environment facilitates the degradation of chlorophyll into brown pyropheophytin, which is primarily responsible for the observed discoloration [65]. In addition, the fibers subjected to alkalization show a more porous surface, while after thermal treatment, they retain their original structure to a greater extent.

### 3.2. Analysis of PLA/Fiber Composites

#### 3.2.1. Optical Microscopy

**Figure 5:**
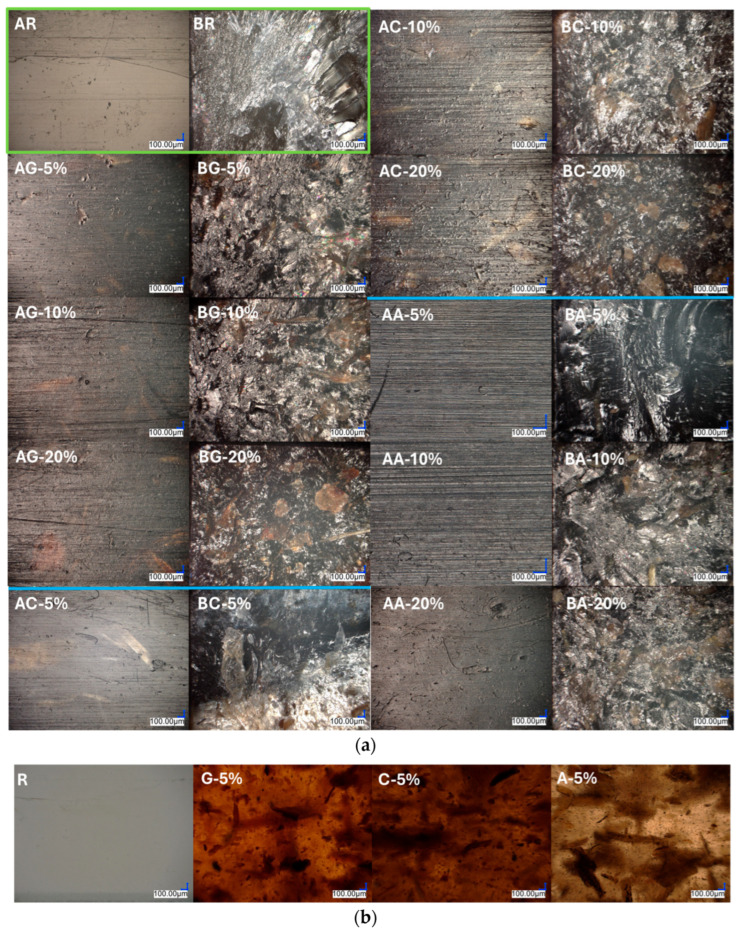
(**a**) Microscopic images of the obtained composites. Image labels consist of two letters and, for modified samples, an indication of the additive concentration (5%, 10%, 20%). The first letter denotes the type of image: A—sample surface, B—fracture surface. The second letter refers to the type of fiber used: R—reference sample, G—raw fibers, C—cooked fibers, A—alkali-treated fibers. (**b**) Microscopic images of fiber dispersion under transmitted light for composites with a 5% filler concentration. R—reference sample, G—raw fibers, C—cooked fibers, A—alkali-treated fibers.

Figure 5a presents optical microscopy images of the obtained composites. The images acquired using transmitted light enabled a preliminary assessment of fiber dispersion in the 5% systems (Figure 5b). Similar to the analysis of the fillers alone, the most pronounced color change toward brown hues is observed in the case of the alkali-treated composite (Figure 5b, A-5%), indicating significant chemical alterations within the fiber structure as a result of the treatment. In this system, a lower concentration of visible filler particles suspended in the matrix was also observed, which may suggest improved dispersion.

Surface images of the fabricated specimens (Figure 5a, series A) indicate that the optimal fiber concentration is 5% for all fiber modifications. However, in the case of the alkali-treated fibers, beneficial effects are also observed at a higher filler content of 10% (Figure 5a, AA-10%). Increasing the filler content beyond these values results in reduced surface homogeneity and increased roughness, which may negatively affect the mechanical properties of the composite.

For the composites containing raw and boiled fibers, larger agglomerates or fiber fragments are visible, which may indicate insufficient adhesion to the matrix and potentially reduce the material’s cohesion. Fracture surface observations (Figure 5a, series B) support these conclusions; in the fracture regions of the composites containing raw and boiled fibers (Figure 5a, BG, BC), numerous poorly bonded fibers are present, in contrast to the samples with alkali-treated fibers (Figure 5a, BA), where the material structure appears more uniform and compact.

#### 3.2.2. Melt Flow Index (MFI)

**Figure 6:**
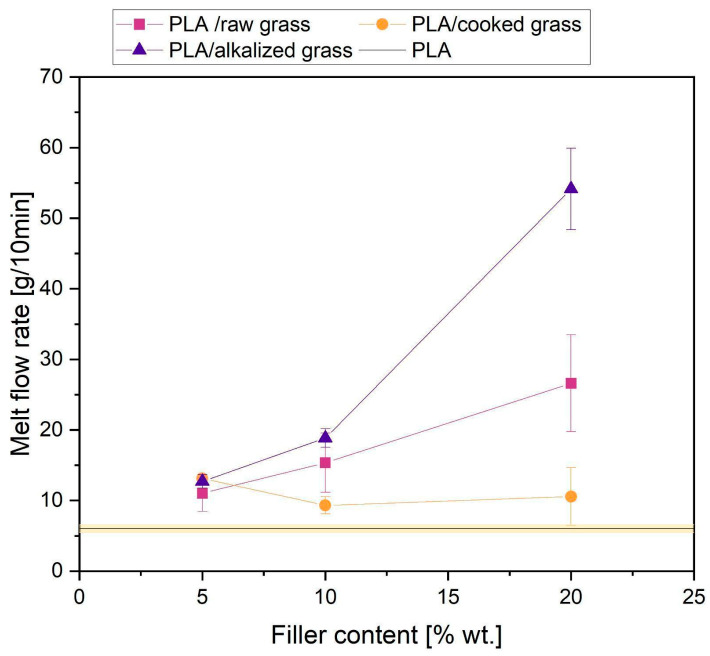
Melt flow index (MFI) of tested samples.

The alkalization process (KOH treatment) removes hemicelluloses, lignin, and other amorphous components, exposing more reactive cellulose and increasing the specific surface area of the fibers. Such modification may contribute to the degradation of PLA during processing, potentially due to alkaline residues retained within the fiber structure. These residues can reduce the molecular weight of the polymer and consequently increase the melt flow index. Additionally, the chemical changes occurring in the lignin structure during alkalization, such as deprotonation of phenolic groups may lead to the formation of low-molecular-weight, surface-active compounds, which can affect the flow behavior of the melt and influence MFI values [66]. Figure 6 illustrates the effect of the natural fiber content on the melt flow index of PLA-based composites. The addition of natural fibers, both raw and cooked/alkalized, leads to an increase in MFI compared to neat PLA (approximately 6–7 g/10 min). Studies have demonstrated that increasing the fiber content in PLA results in higher shear rates during composite processing [67]. This may explain the observed increase in PLA MFI with fiber addition and the further rise at higher filler concentrations. Complex melt flow behavior was observed. In particular, for composites with alkalized grass, a significant increase in MFI was observed; for 10% and 20% filler content, MFI relative to reference PLA increased by 3 times and 9 times, respectively. This behavior is attributed to morphological and chemical changes caused by the treatment, such as surface smoothing and partial degradation of the polymer matrix. The partial degradation was also confirmed by GPC analysis (Section 3.2.3), especially for the samples containing alkalized fibers. Both GPC analysis and MFI measurements indicate that fiber additives, especially after alkalization, affect the molecular structure of PLA, as evidenced by an increase in the polydispersity index (PD). This is evidenced by broadening of the molecular weight distribution and marked increase in MFI. The increase in MFI suggests enhanced flowability, probably due to fiber alignment under shear and reduced melt viscosity. This is consistent with the GPC results, the more degraded the PLA, the lower the viscosity and the higher the MFI. These results are in line with previous studies reporting similar effects in fiber-reinforced PLA systems [21,22,23,68]

#### 3.2.3. Gel Permeation Chromatography (GPC)

**Figure 7:**
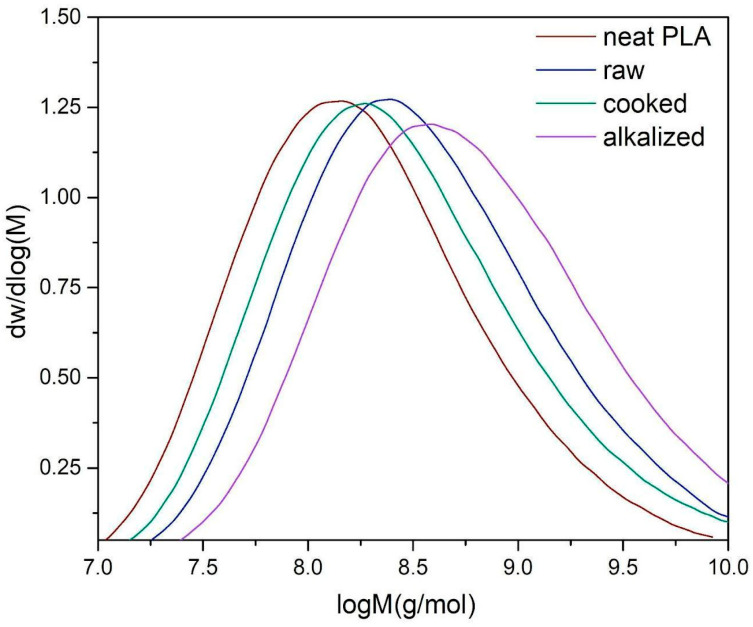
GPC results of PLA and composites.

To assess the impact of the addition of plant fibers and their chemical modification on the structure of the PLA matrix, gel permeation chromatography analysis was conducted. Figure 7 presents the molecular weight distributions (dw/dlogM as a function of logM), and Table 2 summarizes the polydispersity index (PDI) values for the analyzed samples. For the reference sample (neat PLA), a symmetric molecular weight distribution was observed. The incorporation of raw (untreated) Poaceae fibers led to a noticeable broadening of the molecular weight distribution. This effect was further intensified in the sample with thermally pretreated (boiled) fibers. The most pronounced broadening was observed for the composite containing alkalized fibers, indicating the highest degree of distribution heterogeneity among all tested materials. An increase in the polydispersity index was recorded for all fiber-containing samples, with the highest value observed for the alkalized sample (PDI = 1.818), indicating increased heterogeneity in polymer chain lengths. This growing variation in chain length may reflect partial degradation of PLA during processing, particularly in the presence of chemically modified fibers. Such treated fibers may catalyze the degradation of PLA during processing, partly due to the presence of alkaline residues within the fiber structure, which increase the PD coefficient [69].

These findings suggest that the incorporation of plant fibers, especially chemically treated ones, affects the structural stability of PLA, which may influence the final properties of the composite materials.

#### 3.2.4. DSC Results—T_g_, T_cc_, and T_m_ of PLA Composites

**Figure 8:**
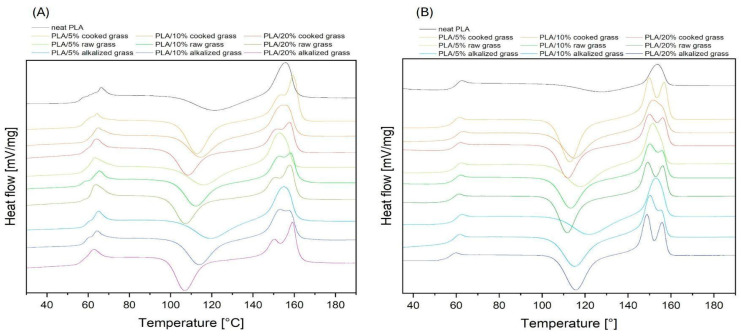
(**A**) DSC curves in the first heating cycle, (**B**) DSC curves in the second heating cycle.

Based on the conducted differential scanning calorimetry analysis, the characteristic phase transition temperatures of semicrystalline PLA-based composites were identified, including the glass transition temperature (T_g_), cold crystallization temperature (T_cc_), and melting temperature (T_m_) (Figure 8). The characteristic values for each formulation are presented in Table 3. The results demonstrate that the incorporation of Poaceae-based fillers, regardless of their type (raw, cooked, or alkalized) leads to a reduction in cold crystallization temperature (T_cc_) compared to neat PLA. For example, the cold crystallization temperature for neat PLA in the second cycle is 127.3 °C, whereas for the PLA/20% raw Poaceae composite, T_cc_ is reduced by as much as 15.6 °C. This effect is consistently observed in both the first and second heating cycles. Moreover, increasing the filler content results in a further decrease in T_cc_ in both cycles. This behavior indicates that the presence of Poaceae fibers leads to earlier crystallization of PLA upon heating [70]. The fibers likely act as nucleating agents, promoting the development of crystalline domains at lower temperatures. In addition to influencing cold crystallization, Poaceae fillers also significantly affect the melting behavior of the composites. Broadening and splitting of the melting endotherms are observed in both the first and second heating cycles. The presence of multiple melting peaks indicates the formation of heterogeneous crystalline structures with varying thermal stability. Such diversity in crystal morphology may also be associated with reorganization or recrystallization processes occurring during heating. This suggests that the fillers, regardless of their processing method, disturb the uniformity of crystallization, leading to the coexistence of crystallites with different morphologies. Overall, these findings underline the dual role of Poaceae-based fillers in PLA composites: they function as nucleating agents that accelerate crystallization kinetics by lowering T_cc_ while simultaneously acting as morphological disruptors that reduce the structural uniformity of the crystalline phase, as seen by the broadened and split melting transitions.

#### 3.2.5. Tensile Strength of PLA Composites with Poaceae-Based Fillers: Influence of Filler Content and Fiber Treatment

**Figure 9:**
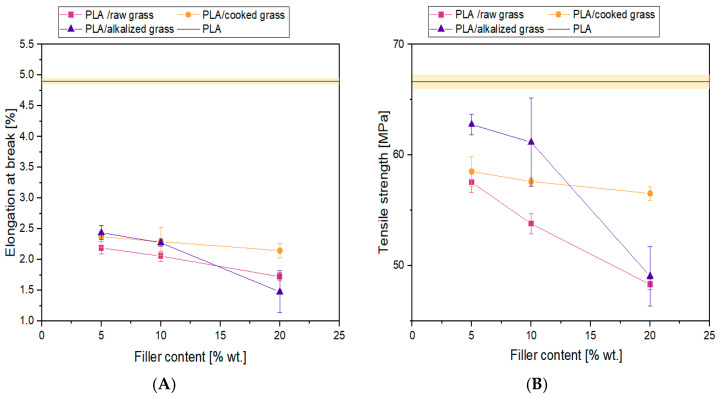
Elongation at break (**A**) and tensile strength (**B**) of the tested samples.

The tensile strength of all tested PLA composites containing Poaceae is presented in Figure 9. The graph includes a reference curve for neat PLA and the tensile strength limit (R_m_) values for the tested composites. In all cases, a decrease in R_m_ was observed compared to the reference material. It was noted that this parameter decreased systematically with increasing filler content, with the most significant reduction observed for raw and alkali-treated Poaceae. For a 20 wt.% filler content, the composite with raw Poaceae showed a decrease in tensile strength of 24.47% and 23.26%, respectively, compared to neat PLA, while the composite containing boiled Poaceae showed a smaller decrease of 15.13%. Among all the analyzed systems, the highest tensile strength values were achieved by the composite containing alkali-treated Poaceae at 5 wt% and 10 wt% filler content, suggesting that at low concentrations these fibers may effectively act as reinforcement, transferring stress and strengthening the composite structure [24,25]. The results of the mechanical properties of the composites can be interpreted in two ways. On the one hand, a deterioration of certain mechanical parameters is observed, which is an undesirable phenomenon.

On the other hand, despite the general decrease in mechanical properties, the obtained values are still higher than those of conventional engineering plastics, such as polypropylene (PP) or polyethylene (PE) [71,72,73,74]. Based on the obtained results, it can be concluded that the applied modification indicates a positive influence on the mechanical strength of the composites. However, it should be emphasized that regardless of the treatment method, the introduction of Poaceae as a filler results in a reduction in tensile strength compared to pure PLA. In the case of boiling, it is assumed that compounds such as proteins, pectins, or polysaccharides are removed from the fiber structure. Since pectins act as natural structural binders, their removal leads to the disintegration of the structure and weakening of mechanical properties. Moreover, breaking the fibers into smaller cellulose units allows for better wetting by the polymer matrix. As a result, composites with alkali-treated fibers at a low content (5 and 10 wt%) show higher tensile strength than those with boiled fibers. However, at a higher filler content (20 wt%), the composite with boiled Poaceae achieves better mechanical results. At lower concentrations of alkali-treated fibers, better dispersion in the polymer matrix is observed, which translates into improved mechanical properties of the composites. Conversely, at higher fiber concentrations, there is a tendency for agglomeration, which may act as structural defects (notches), leading to deterioration of the mechanical properties of the composite. An important aspect affecting the mechanical properties of composites is also the fiber size, particularly their diameter. According to the literature, better mechanical effects are achieved using fibers with smaller diameters [75,76,77]. Fibers with large cross-sections tend to develop internal cracking, which, despite good adhesion to the matrix, worsens the structural integrity of the entire composite. It should also be noted that the sample preparation method has a significant impact on tensile strength and elongation at break. In this study, samples were produced by injection molding, which causes orientation of the particles along the material flow direction. For samples where the longer axis coincided with the flow direction, the presence of short fibers with limited adhesion to the matrix further deteriorated the mechanical properties. Microscopic observations confirmed generally poor fiber-to-matrix adhesion, due to the presence of lignin, fats, and surface waxes, which limit effective stress transfer between the fiber and the PLA matrix [26], making this one of the key factors negatively affecting the composite properties. Similar trends were observed for the elongation at break results (Figure 9); hence, a detailed commentary in this section has been omitted. It is worth noting, however, that at lower concentrations (5 and 10 wt%), similar elongation values were recorded for composites containing boiled and alkali-treated Poaceae. The least favorable deformation values were observed for the composite containing 20% chemically modified fibers. This may be due to the presence of nucleation centers and an increase in local crystalline phases, leading to increased brittleness of the material.

**Figure 10:**
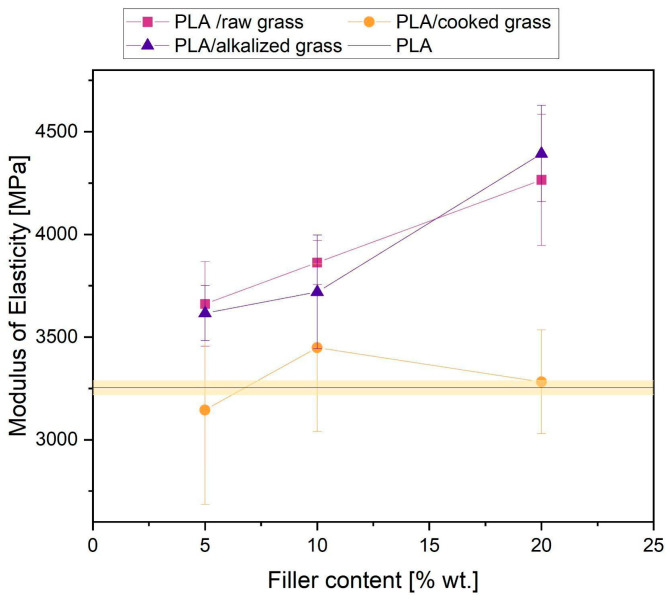
Modulus of elasticity of tested samples.

The modulus of elasticity of all tested PLA composites with Poaceae addition showed an increasing trend with increasing filler content, with the strongest enhancement effect observed for composites containing alkalized and raw Poaceae (Figure 10). For a concentration of 20 wt.%, the increase in modulus relative to neat PLA was 35% (alkalized) and 31% (raw Poaceae), respectively, suggesting that, with the right degree of filler, these fibers can effectively stiffen the polymer matrix. The lowest modulus values among the modified composites were recorded for the cooked Poaceae system at a low filler content (5 wt.%), which may be related to the insufficient stiffness of the fibers subjected to prolonged heat treatment or their inhomogeneous dispersion.

Table 4 presents the values of elongation at break, tensile strength, and modulus of elasticity for the fabricated composites.

#### 3.2.6. Impact Strength of PLA Composites with Poaceae Fillers

**Figure 11:**
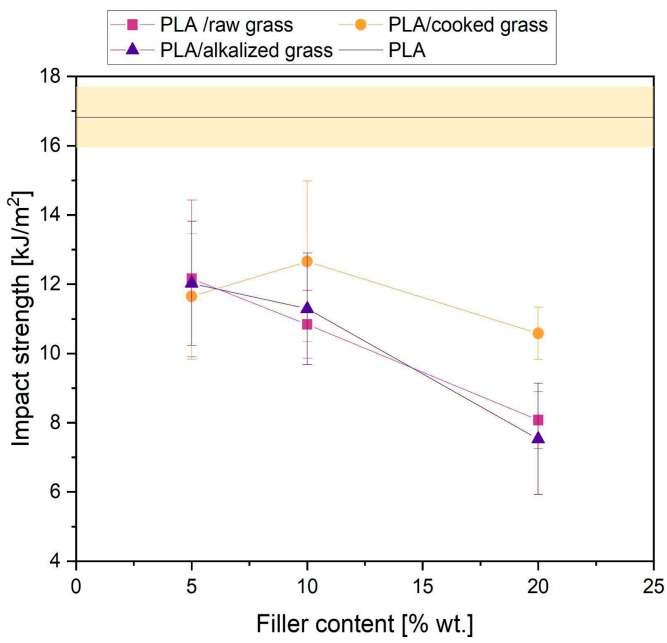
Impact strength of tested samples.

The conducted studies revealed that the addition of Poaceae as a filler leads to a reduction in the impact strength of composites compared to neat PLA (Figure 11). At a filler content of 5 wt.%, regardless of the type of fiber treatment applied, very similar impact strength values were obtained, ranging between 11.6–12.1 kJ/m^2^. This represents a decrease of approximately 29% compared to the reference sample (16.8 kJ/m^2^). As the fiber content increased to 10 wt.% and 20 wt.%, a further and more pronounced reduction in impact strength was observed, particularly in the composites containing alkalized and untreated Poaceae fibers. For the 20 wt.% content, the impact strength dropped to 7.7–8.5 kJ/m^2^, indicating a reduction of more than 50% relative to neat PLA. Such a significant decline in impact resistance may result from poor interfacial adhesion and the presence of structural defects, such as fiber agglomerates, which act as crack initiation sites. Among all the composites tested, the best impact properties were observed for samples containing Poaceae subjected to boiling, especially at a 10 wt% filler content. This improvement may be attributed to more homogeneous fiber dispersion within the polymer matrix and the partial removal of components such as lignin, fats, or other surface-active substances that negatively affect interfacial adhesion. The boiling process may also have led to fiber structure loosening, increasing their compatibility with the polymer phase and thereby mitigating the negative impact of the filler on impact resistance. These findings suggest that an appropriately selected fiber treatment can alleviate the adverse effects of natural fillers on the impact strength of PLA-based composites, as also noted in the literature [70].

#### 3.2.7. Flexural Strength and Flexural Modulus Analysis

**Figure 12:**
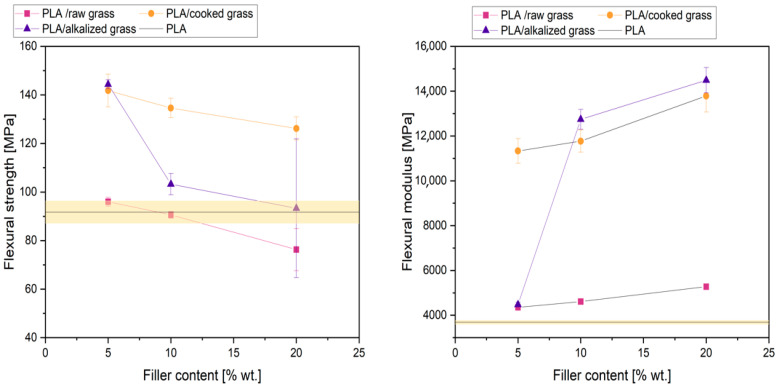
Flexural strength and flexural modulus of tested samples.

The conducted research showed that among all the produced composites, the best mechanical performance in terms of flexural strength was observed for systems containing alkalized and boiled Poaceae fibers. Figure 12 presents the flexural strength and flexural modulus of the tested samples. Composites with untreated Poaceae exhibited flexural strength values comparable to neat PLA (98 MPa) at filler contents of 5 wt% and 10 wt%. In the case of 20 wt% of untreated Poaceae, a 17% decrease in flexural strength was recorded compared to the reference sample, which may indicate matrix overloading with a filler exhibiting poor interfacial adhesion, resulting in reduced structural integrity of the composite. The highest flexural strength values were obtained for composites with 5 wt% of alkalized (141.8 MPa) and boiled Poaceae (144.4 MPa), representing increases of 54% and 57%, respectively, compared to neat PLA. In all analyzed cases, increasing the filler content resulted in a systematic decline in flexural strength, indicating a loss of matrix phase continuity at higher fiber loadings. The conclusions drawn from the tensile strength analysis are consistent with the results of flexural strength testing. The alignment of the fibers and the structural fragmentation resulting from boiling positively influenced the flexural properties. A more uniform fiber dispersion and a layered arrangement within the polymer matrix improved the ability of the material to transfer mechanical loads acting perpendicular to the fiber orientation. Additionally, the finer fiber structure reduced the likelihood of internal cracking. In contrast, composites with alkalized fibers—despite exhibiting better adhesion to the polymer matrix—contained larger fiber fractions that were more prone to internal fracture, which may explain the less favorable flexural performance. The obtained flexural modulus values further support these observations (Figure 12). All modified systems demonstrated higher flexural moduli than the reference PLA sample (3686 ± 85 MPa), confirming the stiffening effect induced by the presence of lignocellulosic fibers. The greatest increases were observed in composites with 20 wt.% of alkalized (14,487 MPa) and boiled Poaceae (13,789 MPa), corresponding to 393% and 374% of the reference value, respectively. This effect can be attributed to the high stiffness of the natural fibers, which, in combination with the ductile PLA matrix, significantly enhanced the material’s resistance to elastic deformation. It is also worth noting that favorable values of flexural modulus were achieved with boiled fibers already at 5 wt.% content, suggesting better compatibility with the matrix structure. In contrast, for alkalized fibers, enhanced modulus values were observed only at higher filler contents, likely due to the presence of larger fiber fractions with a greater tendency to deform.

## 4. Conclusions

The use of Poaceae fibers as a filler in polylactide (PLA)-based composites has a multifaceted impact on the mechanical, structural, and processing properties of the resulting materials. The addition of fibers reduced the cold crystallization temperature (T_cc_) by up to 15.6 °C, confirming their nucleating effect. The melt flow index increased with the fiber content, particularly in systems with alkalized fibers, indicating reduced viscosity. The tensile strength decreased with increasing fiber content, with the most substantial reduction observed at 20 wt.%. Despite this, composites with 5–10 wt.% boiled or alkalized fibers showed improved flexural strength, exceeding neat PLA by over 50%. The modulus of elasticity increased proportionally with the filler content, reaching 4393 MPa for 20 wt.% alkalized fibers. The results confirm that Poaceae fibers can serve as functional fillers in PLA composites, improving stiffness. However, the mechanical performance is sensitive to both the fiber type and content. Composites containing 5–10 wt.% treated fibers provided the most favorable balance between strength, stiffness, and processability. These findings provide a basis for the further development of bio-based PLA composites with tailored properties for engineering applications. The obtained results confirm the viability of using Poaceae fibers as an environmentally friendly filler for PLA. Proper selection of the modification parameters and fiber content enables the effective management of the trade-off between various mechanical properties, supporting the development of biodegradable composite materials with improved functional performance. These results align with the growing interest in biodegradable composites as a sustainable alternative to synthetic materials.

## Figures and Tables

**Table 1:** TGA results.

	T1 [°C]	Mass Change [%]	Onset [°C]	T2 [°C]	Mass Change [%]	Residual Mass [%]
Raw	58.1	7.16	249.9	281.5/337.3	60.82	26.36
Cooked	54.7	4.18	271.3	311.3/362.3	67.88	14.45
Alkalized	59.2	3.85	275.2	354.5	71.12	14.99

**Table 2:** Polydispersity coefficient.

Sample	PD
Neat PLA	1.680
Raw	1.692
Cooked	1.762
Alkalized	1.818

**Table 3:** Characteristic phase transition temperatures based on DSC analysis.

	T_g_ [°C]		T_cc_ [°C]		T_m_ [°C]	
Cycle	1	2	1	2	1	2
Neat PLA	66.4	62.2	121.3	127.3	155.7	153.8
PLA/5% Raw Poaceae	63.3	61.9	115.9	118.0	152.9	151.6
PLA/10% Raw Poaceae	58.1	61.7	112.3	113.3	152.9/158.0	150.2/155.6
PLA/20% Raw Poaceae	63.4	60.8	107.2	111.7	151.0/157.8	149.2/156.1
PLA/5% Cooked Poaceae	64.2	61.1	112.8	112.9	153.8/159.4	149.9/156.7
PLA/10% Cooked Poaceae	64.7	62.7	114.4	115.0	155.8	152.0
PLA/20% Cooked Poaceae	63.6	61.6	108.4	111.9	151.8/157.8	149.9/156.2
PLA/5% Alkalized Poaceae	64.9	62.2	119.5	121.7	155.2	153.0
PLA/10% Alkalized Poaceae	64.2	61.4	114.0	115.1	153.3/157.3	150.2/155.2
PLA/20% Alkalized Poaceae	62.6	59.5	106.9	115.7	150.4/159.1	148.8/155.9

**Table 4:** Elongation at break (%), tensile strength (MPa), and modulus of elasticity (MPa) of the obtained composites.

	Elongation at Break [%]	st. d. [-]	Tensile Strength [MPa]	st. d. [-]	Modulus of Elasticity [MPa]	st. d. [-]
Neat PLA	4.90	0.05	66.6	0.6	3253	34
PLA/5% Raw Poaceae	2.19	0.10	57.5	0.9	3661	206
PLA/10% Raw Poaceae	2.06	0.08	53.8	0.9	3863	107
PLA/20% Raw Poaceae	1.72	0.07	48.3	0.5	4266	320
Poaceae PLA/5% Cooked	2.37	0.17	58.5	1.3	3145	461
Poaceae PLA/10% Cooked Poaceae	2.29	0.23	57.6	0.3	3448	408
PLA/20% Cooked Poaceae	2.15	0.12	56.5	0.6	3282	252
Poaceae PLA/5% Alkalized	2.44	0.11	62.8	0.9	3616	134
PLA/10% Alkalized Poaceae	2.28	0.06	61.2	4.0	3720	277
PLA/20% Alkalized Poaceae	1.48	0.34	49.0	2.7	4393	235

## Data Availability

The original contributions presented in this study are included in the article. Further inquiries can be directed to the corresponding authors.

## References

[1] Geyer R., Jambeck J.R., Law K.L. (2017). Production, use, and fate of all plastics ever made. Sci. Adv..

[2] Barnes D.K.A., Galgani F., Thompson R.C., Barlaz M. (2009). Accumulation and fragmentation of plastic debris in global environments. Philos. Trans. R. Soc. Lond. B Biol. Sci..

[3] Peelman N., Ragaert P., De Meulenaer B., Adons D., Peeters R., Cardon L., Van Impe F., Devlieghere F. (2013). Application of bioplastics for food packaging. Trends Food Sci. Technol..

[4] Gołębiewski J., Gibas E., Malinowski R. Wybrane Polimery Biodegradowalne—Otrzymywanie, Właściwości, Zastosowanie. https://yadda.icm.edu.pl/baztech/element/bwmeta1.element.baztech-article-BAT7-0013-0002.

[5] EUBIO_Admin Market. https://www.european-bioplastics.org/market/.

[6] Jamshidian M., Tehrany E.A., Imran M., Jacquot M., Desobry S. (2010). Poly—Lactic Acid: Production, Applications, Nanocomposites, and Release Studies. Compr. Rev. Food Sci. Food Saf..

[7] Kale G., Kijchavengkul T., Auras R., Rubino M., Selke S.E., Singh S.P. (2007). Compostability of Bioplastic Packaging Materials: An Overview. Macromol. Biosci..

[8] Garlotta D. (2001). A Literature Review of Poly(Lactic Acid). J. Polym. Environ..

[9] Zuza E., Meaurio E., Sarasua J.-R. (2016). Biodegradable Polylactide-Based Composites. Composites from Renewable and Sustainable Materials.

[10] Ramanjaneyulu B., Venkatachalapathi N., Prasanthi G. (2021). Thermal and Mechanical Properties of PLA/ABS/TCS Polymer Blend Composites. J. Inst. Eng. (India) Ser. C.

[11] El-Taweel S.H., Abboudi M. (2019). Nonisothermal crystallization kinetics of PLA/nanosized YVO_4_ composites as a novel nucleating agent. J. Appl. Polym. Sci..

[12] Brząkalski D., Sztorch B., Frydrych M., Pakuła D., Dydek K., Kozera R., Boczkowska A., Marciniec B., Przekop R.E. (2020). Limonene Derivative of Spherosilicate as a Polylactide Modifier for Applications in 3D Printing Technology. Molecules.

[13] Kulinski Z., Piorkowska E., Gadzinowska K., Stasiak M. (2006). Plasticization of Poly(l–lactide) with Poly(propylene glycol). Biomacromolecules.

[14] Marin N.M. (2025). Green Chemistry Applications Using Complexing Materials for Water Treatment. Polymers.

[15] Abraha K.G., Debeli D.K., Ghani M.U., Zhou B., Tesfahunegn A.A., Guo J. (2024). Enhancing enset fiber-reinforced polylactic acid composites through different surface treatments for emerging applications. Polym. Compos..

[16] Sholokhova A., Varžinskas V., Rutkaitė R. (2024). Valorization of Agro-waste in Bio-based and Biodegradable Polymer Composites: A Comprehensive Review with Emphasis on Europe Perspective. Waste Biomass Valorization.

[17] Vinod B., Murthy B.V., Ramesh V., Venkataramana P. (2025). Extraction and Performance of Novel Lignocellulose from Agro-Wastes: Synergistic Integration of Waste into Ecofriendly Polymer Composites. Key Eng. Mater..

[18] Członka S., Strąkowska A., Kairytė A. (2020). Effect of walnut shells and silanized walnut shells on the mechanical and thermal properties of rigid polyurethane foams. Polym. Test..

[19] Ilyas R.A., Zuhri M.Y.M., Aisyah H.A., Asyraf M.R.M., Hassan S.A., Zainudin E.S., Sapuan S.M., Sharma S., Bangar S.P., Jumaidin R. (2022). Natural Fiber-Reinforced Polylactic Acid, Polylactic Acid Blends and Their Composites for Advanced Applications. Polymers.

[20] Guo R., Azaiez J., Bellehumeur C. (2005). Rheology of fiber filled polymer melts: Role of fiber-fiber interactions and polymer-fiber coupling. Polym. Eng. Sci..

[21] Liang J., Li R., Tjong S. (1999). Effects of pressure and temperature on the melt density and the melt flow rate of LDEP and glass bead-filled LDPE composite. J. Mech. Work. Technol..

[22] Lafranche E., Martins C.I., Oliveira V.M., Krawczak P. (2013). Prediction of Tensile Properties of Injection Moulding Flax Fibre Reinforced Polypropylene from Morphology Analysis. Key Eng. Mater..

[23] Lee C.H., Sapuan S.M., Lee J.H., Hassan M.R. (2016). Melt volume flow rate and melt flow rate of kenaf fibre reinforced Floreon/magnesium hydroxide biocomposites. SpringerPlus.

[24] Li X., Tabil L.G., Panigrahi S. (2007). Chemical Treatments of Natural Fiber for Use in Natural Fiber-Reinforced Composites: A Review. J. Polym. Environ..

[25] Pickering K.L., Aruan Efendy M.G., Le T.M. (2016). A review of recent developments in natural fibre composites and their mechanical performance. Compos. Part A Appl. Sci. Manuf..

[26] Kumar G., Ohkubo T., Hono K. (2009). Effect of melt temperature on the mechanical properties of bulk metallic glasses. J. Mater. Res..

[27] Gunti R., Prasad A.R., Gupta A. (2016). Mechanical and degradation properties of natural fiber-reinforced PLA composites: Jute, sisal, and elephant grass. Polym. Compos..

[28] George J., Sreekala M.S., Thomas S. (2001). A review on interface modification and characterization of natural fiber reinforced plastic composites. Polym. Eng. Sci..

[29] Faruk O., Bledzki A.K., Fink H.-P., Sain M. (2012). Biocomposites reinforced with natural fibers: 2000–2010. Prog. Polym. Sci..

[30] Battegazzore D., Noori A., Frache A. (2018). Natural wastes as particle filler for poly(lactic acid)-based composites. J. Compos. Mater..

[31] Salasinska K., Polka M., Gloc M., Ryszkowska J. (2016). Natural fiber composites: The effect of the kind and content of filler on the dimensional and fire stability of polyolefin-based composites. Polimery.

[32] Song X., Song P., Qin H., Meng H. (2020). Fused Deposition Modeling of Montmorillonite Modified Poly (Lactic Acid)/Sunflower Seed Husk Composites. IOP Conference Series: Materials Science and Engineering.

[33] Salasinska K., Barczewski M., Borucka M., Górny R.L., Kozikowski P., Celiński M., Gajek A. (2019). Thermal Stability, Fire and Smoke Behaviour of Epoxy Composites Modified with Plant Waste Fillers. Polymers.

[34] Fayzullin I., Gorbachev A., Volfson S., Serikbayev Y., Nakyp A., Akylbekov N. (2024). Composite Material Based on Polypropylene and Modified Natural Fillers. Polymers.

[35] Kuram E. (2020). UV and thermal weathering of green composites: Comparing the effect of different agricultural waste as fillers. J. Compos. Mater..

[36] Zakrzewska P., Zygmunt-Kowalska B., Pielichowska K., Nowicka-Dunal K., Telejko T., Kuźnia M. (2024). Effect of the silanization process on the fire resistance and thermal properties of closed-cell foams with sunflower husk ash. Ind. Crop. Prod..

[37] Keller A. (2003). Compounding and mechanical properties of biodegradable hemp fibre composites. Compos. Sci. Technol..

[38] Kausar A., Ahmad I. (2023). Hemp Fibres: Essentials, Composites or Nanocomposites and Technical Applications. Nano-Horizons J. Nanosci. Nanotechnol..

[39] Ari A., Karahan M., Kopar M., Ahrari M., Khan R.M.W.U., Hussain M. (2023). Comparative analysis of natural fibres characteristics as composite reinforcement. Ind. Textila.

[40] Alao P.F., Press R., Ruponen J., Mikli V., Kers J. (2023). Influence of Protic Ionic Liquid-Based Flame Retardant on the Flammability and Water Sorption of Alkalized Hemp Fiber-Reinforced PLA Composites. Polymers.

[41] Aguado R.J., Bastida G.A., Espinach F.X., Llorens J., Tarrés Q., Delgado-Aguilar M., Mutjé P. (2023). Comparative Study on the Stiffness of Poly(lactic acid) Reinforced with Untreated and Bleached Hemp Fibers. Polymers.

[42] Benhadou B., Haddout A., Benhadou M., Ourchid H. (2016). Study of Thermorheological Behavior of Polypropylene Composites Reinforced with Short Hemp Fibers During Industrial Injection Molding Process. Int. J. Mech. Eng. Technol. (IJMET).

[43] Rajesh G., Revuri A., Arekapudi M.S., Dbm G.R. (2019). Evaluating Tensile Properties of Phragmites Karka Fibre Reinforced Polyester Composites. Mater. Today Proc..

[44] Ortega Z., Suárez L., Kelly-Walley J., Hanna P.R., McCourt M., Millar B. (2024). Use of Pressure in Rotational Molding to Reduce Cycle Times: Comparison of the Thermomechanical Behavior of Rotomolded Reed/Polyethylene Composites. J. Compos. Sci..

[45] Honoré M., Pimbert S., Lecompte T. (2020). Characterisation of plant flours for biocomposite applications focussing on Phragmites australis properties. Biosyst. Eng..

[46] Suárez L., Barczewski M., Kosmela P., Marrero M.D., Ortega Z. (2022). Giant Reed (*Arundo donax* L.) Fiber Extraction and Characterization for Its Use in Polymer Composites. J. Nat. Fibers.

[47] Ganesan K., Kailasanathan C., Rajini N., Ismail S.O., Ayrilmis N., Mohammad F., Al-Lohedan H.A., Tawfeek A.M., Issa Z.A., Aldhayan D.M. (2021). Assessment on hybrid jute/coir fibers reinforced polyester composite with hybrid fillers under different environmental conditions. Constr. Build. Mater..

[48] Kim B.S., Nguyen M.H., Hwang B.S., Lee S. (2008). Effect of plasma treatment on the mechanical properties of natural fiber/PP composites. WIT Trans. Built Environ..

[49] Hasan K.M.F., Horváth P.G., Bak M., Alpár T. (2021). A state-of-the-art review on coir fiber-reinforced biocomposites. RSC Adv..

[50] Adeniyi A.G., Onifade D.V., Ighalo J.O., Adeoye A.S. (2019). A review of coir fiber reinforced polymer composites. Compos. Part B Eng..

[51] Kakou C.A., Essabir H., Bensalah M.-O., Bouhfid R., Rodrigue D., Qaiss A. (2015). Hybrid composites based on polyethylene and coir/oil palm fibers. J. Reinf. Plast. Compos..

[52] Reddy N. (2019). Sustainable Applications of Coir and Other Coconut By-products.

[53] Ramirez C., Agaliotis E., Pettarin V. (2024). Fracture toughness and overall characterization of PLA based biocomposites with natural fibers: A comparative study. Polymer.

[54] Gregorova A., Hrabalova M., Kovalcik R., Wimmer R. (2010). Surface modification of spruce wood flour and effects on the dynamic fragility of PLA/wood composites. Polym. Eng. Sci..

[55] Jubinville D., Tzoganakis C., Mekonnen T.H. (2022). Recycled PLA—Wood flour based biocomposites: Effect of wood flour surface modification, PLA recycling, and maleation. Constr. Build. Mater..

[56] Bhayana M., Singh J., Sharma A., Gupta M. (2023). A review on optimized FDM 3D printed Wood/PLA bio composite material characteristics. Mater. Today Proc..

[57] (2019). Plastics—Test Specimens.

[58] (2020). Plastics—Determination of tensile properties—Part 1: General principles.

[59] (2019). Plastics—Determination of flexural properties.

[60] (2006). Plastics - Determination of the melt mass-flow rate (MFR) and the melt volume-flow rate (MVR) of thermoplastics (ISO 1133:2005).

[61] Tiwari Y.M., Sarangi S.K. (2022). Characterization of raw and alkali treated cellulosic Grewia Flavescens natural fiber. Int. J. Biol. Macromol..

[62] Zhou X.-L., Liu Z.-M., Kiss J., Sloan D.W., White J.M. (1995). Surface Chemistry of Chloroiodomethane, Coadsorbed with H and O, on Pt(111). J. Am. Chem. Soc..

[63] Reddy K.O., Maheswari C.U., Shukla M., Rajulu A. (2012). Chemical composition and structural characterization of Napier grass fibers. Mater. Lett..

[64] Reza S., Islam S.N., Afroze S., Abu Bakar M.S., Taweekun J., Azad A.K. (2020). Data on FTIR, TGA – DTG, DSC of invasive pennisetum purpureum grass. Data Brief.

[65] Pozo M.B.-D., Gallardo-Guerrero L., Gandul-Rojas B. (2020). Influence of Alkaline Treatment on Structural Modifications of Chlorophyll Pigments in NaOH—Treated Table Olives Preserved without Fermentation. Foods.

[66] Helander M., Theliander H., Lawoko M., Henriksson G., Zhang L., Lindström M.E. (2013). Fractionation of Technical Lignin: Molecular Mass and pH Effects. BioResources.

[67] Arias A., Sojoudiasli H., Heuzey M.-C., Huneault M.A., Wood-Adams P. (2017). Rheological study of crystallization behavior of polylactide and its flax fiber composites. J. Polym. Res..

[68] Běhálek L., Maršálková M. Study of Crystallization of Polylactic Acid Composites and Nanocomposites with Natural Fibres by DSC Method. 2018. https://www.researchgate.net/publication/288205389_Study_of_crystallization_of_polylactic_acid_composites_and_nanocomposites_with_natural_fibres_by_DSC_method.

[69] Ma C., Guo Q., Mo J., Lin F., Chen J., Cao D., Xie W., He K., Liu X., Xie G. (2023). Monitoring and kinetic study of alkaline hydrolysis of polylactic acid fibrous membrane via aggregation-induced emission (AIE) technique. Surfaces Interfaces.

[70] Dittenber D.B., GangaRao H.V. (2012). Critical review of recent publications on use of natural composites in infrastructure. Compos. Part A Appl. Sci. Manuf..

[71] Yang B.-X., Shi J.-H., Pramoda K., Goh S.H. (2008). Enhancement of the mechanical properties of polypropylene using polypropylene-grafted multiwalled carbon nanotubes. Compos. Sci. Technol..

[72] Ellis T.S., D’Angelo J.S. (2003). Thermal and mechanical properties of a polypropylene nanocomposite. J. Appl. Polym. Sci..

[73] Yang B., Pramoda K.P., Xu G.Q., Goh S.H. (2007). Mechanical Reinforcement of Polyethylene Using Polyethylene-Grafted Multiwalled Carbon Nanotubes. Adv. Funct. Mater..

[74] Cho K., Lee B.H., Hwang K., Lee H., Choe S. (1998). Rheological and mechanical properties in polyethylene blends. Polym. Eng. Sci..

[75] Qi Z., Wang B., Sun C., Yang M., Chen X., Zheng D., Yao W., Chen Y., Cheng R., Zhang Y. (2022). Comparison of Properties of Poly(Lactic Acid) Composites Prepared from Different Components of Corn Straw Fiber. Int. J. Mol. Sci..

[76] Arao Y., Fujiura T., Itani S., Tanaka T. (2015). Strength improvement in injection-molded jute-fiber-reinforced polylactide green-composites. Compos. Part B Eng..

[77] Madueke C.I., Mbah O.M., Umunakwe R. (2022). A review on the limitations of natural fibres and natural fibre composites with emphasis on tensile strength using coir as a case study. Polym. Bull..

